# Drinking Water Quality Guidelines across Canadian Provinces and Territories: Jurisdictional Variation in the Context of Decentralized Water Governance

**DOI:** 10.3390/ijerph110504634

**Published:** 2014-04-25

**Authors:** Gemma Dunn, Karen Bakker, Leila Harris

**Affiliations:** 1 Program on Water Governance, University of British Columbia, 439-2202 Main Mall, Vancouver, BC, V6T 1Z4, Canada; E-Mails: Karen.bakker@ubc.ca (K.B.); lharris@ires.ubc.ca (L.H.); 2 Department of Geography, University of British Columbia, 120-1984 West Mall, Vancouver, BC, V6T 1Z2, Canada; 3 Institute of Resources Environment and Sustainability, 421-2202 Main Mall, Vancouver, BC, V6T 1Z4, Canada

**Keywords:** drinking water guidelines, drinking water standards, decentralized water governance, water policy, heterogeneity, Canada

## Abstract

This article presents the first comprehensive review and analysis of the uptake of the Canadian Drinking Water Quality Guidelines (CDWQG) across Canada’s 13 provinces and territories. This review is significant given that Canada’s approach to drinking water governance is: (i) highly decentralized and (ii) discretionary. Canada is (along with Australia) only one of two Organization for Economic Cooperation and Development (OECD) member states that does not comply with the World Health Organization’s (WHO) recommendation that all countries have national, legally binding drinking water quality standards. Our review identifies key differences in the regulatory approaches to drinking water quality across Canada’s 13 jurisdictions. Only 16 of the 94 CDWQG are consistently applied across all 13 jurisdictions; five jurisdictions use voluntary guidelines, whereas eight use mandatory standards. The analysis explores three questions of central importance for water managers and public health officials: (i) should standards be uniform or variable; (ii) should compliance be voluntary or legally binding; and (iii) should regulation and oversight be harmonized or delegated? We conclude with recommendations for further research, with particular reference to the relevance of our findings given the high degree of variability in drinking water management and oversight capacity between urban and rural areas in Canada.

## 1. Introduction

Threats to drinking water across Canada and internationally have drawn attention to the importance of governance in safeguarding drinking water and public health [1,2,3,4,5,6,7]. Within this broader debate, one key issue is the allocation of responsibility for standard setting for drinking water. Drawing on a case study of Canada, this paper analyzes three issues of central importance for water managers and public health officials: (i) should standards be uniform or variable; (ii) should compliancy be voluntary or legally binding; and (iii) should regulation and oversight be harmonized or delegated? These questions are of particular interest internationally given the fact that greater decentralization and subsidiarity have been recent trends in water governance, despite the recommendation by the World Health Organization for national legally binding, uniform standards. 

To address these concerns, the article presents the first comprehensive review and evaluation of the uptake and application of Canadian Drinking Water Quality Guidelines (CDWQG) across Canada’s 13 jurisdictions: 10 provinces and three territories. (Provinces and territories are very similar to American or Australian states. The main difference between provinces and territories is that the former (located in the more populous south of the country) receive power and authority directly from Canada’s Constitution Act of 1867, whereas the latter (located in the sparsely populated north of the country) are granted somewhat more limited mandates and powers from the federal government). This review is significant given Canada’s highly decentralized approach to drinking water governance. Canada is the only Group of Eight (G8) country and (along with Australia) only one of two Organization for Economic Cooperation and Development (OECD) member states that does not have legally enforceable drinking water quality standards at the national level—despite “best practice” World Health Organization (WHO) recommendations [3,8]. Thus, Canada is a “natural laboratory” in which to study the questions raised above, as standards are variable across jurisdictions, regulation is characterized by a combination of harmonization and delegation, and only eight jurisdictions have decided to adopt legally binding standards, while others rely on voluntary approaches. The process of drinking water quality standard-setting in Canada reflects the country’s highly decentralized approach to water governance. Canada is unique amongst industrialized countries, notably with respect to the relatively passive role taken by the federal government in matters of drinking water regulation—in contrast to other federations, such as the USA and European Union, which have a higher degree of consistency across their respective territories, and unlike Canada, mandate legally binding, uniform drinking water quality standards. 

In Canada, decentralization arises as a result of the constitutional division of powers between scales of government. As a result, water management is shared by the federal and provincial governments. The federal government’s water responsibilities include navigable waters, fisheries and transboundary waters. Canada’s ten provinces, which derive their authority from the 1867 Constitution Act, have constitutional responsibility for fresh water, natural resources, and health. The three territories (Northwest Territories, Nunavut, and Yukon), differ in that their power and mandates do not derive from the constitution, but rather are delegated under the authority of Canada’s Parliament—although the Government of Canada is increasingly transferring province-like powers to territorial governments. An attempt at harmonization of drinking water quality has been made through a non-binding federal process: the Federal-Provincial-Technical Committee on Drinking Water (FPTCDW). This federal agency, with membership from the provinces and territories, develops the Canadian Drinking Water Quality Guidelines. Since the national guidelines are voluntary and non-enforceable, Canadian jurisdictions have the autonomy to decide whether or not to adopt the CDWQG, to what extent they apply the guidelines, and whether to make them legally enforceable. In short, it is the jurisdictions that have ultimate responsibility for formulating their own drinking water standards, and ensuring that public health and related goals are met [3,9]. As a result, Canada is one of the few examples whereby drinking-water quality management is not standardized across the country. As such, two key governance issues raised by the current situation in Canada include: (i) the high degree of voluntarism and (ii) delegation of responsibility (or decentralization of authority). 

What are the implications of this decentralization of governance, and associated variation in standards? What can be learned from a careful evaluation of the Canadian case in terms of the variation that this framework has produced? Both within Canada and internationally, some experts argue that greater harmonization and formal regulation (and a lower degree of voluntarism and subsidiarity) is required [10,11,12,13,14]. In contrast, others argue in favor of decentralization, advocate subsidiarity, and assert that public health protection will be improved by greater ‘system knowledge’ and contextually specific approaches, rather than formal and standardized regulations [2,15,16]. Proponents of decentralization also point to Canada’s size and geography, arguing that heterogeneity in governance may be functional (to some degree), because it permits adaptation of regulations to local biophysical conditions. However, critics have argued that that Canada’s decentralized regulatory model and nonbinding drinking water quality guidelines foster inequities, with some Canadians being protected by more stringent rules than others, particularly First Nation and rural communities [10,17,18,19,20,21]. In addition, it is argued that the decentralized approach has impacted data collection and quality, which in turn has created its own set of problems. In summary, the literature suggests that there may be both positive and negative aspects of decentralized water governance [22,23,24]. 

This paper contributes to these debates by providing a systematic study of variation in drinking water standards and guidelines. No prior systematic study of this topic in Canada has been published in the scholarly literature. Our research addresses this gap by compiling an inventory of drinking water quality parameters used in Canadian provinces and territories (the first of its kind); analyzing the extent of variation; identifying the baseline of drinking water parameters applied uniformly across provinces and territories, and highlighting some of the implications, for Canada and for the broader debates as noted. 

## 2. Method

Data was compiled through publicly available information sources including government agency websites, reports and legislation. Each province and territory drinking water guideline/standard was compared against the 2012 Canadian Drinking Water Quality Guidelines, which is comprises of 94 chemical, physical, microbial, and radiological parameters. These parameters include health considerations (maximum acceptable concentrations (MAC)), aesthetic considerations (aesthetic objectives (AO)), and operational guideline values (operational guidance (OG)) [25]. 

The analysis includes five categories:

(1) The province/territory uses exactly the same parameter value as the CDWQG;
(2) The parameter value used by the province/territory is less stringent than the CDWQG;
(3) The parameter value used by the province/territory is more stringent than the CDWQG;
(4) The province/territory uses a parameter that is not listed under the CDWQG;
(5) A CDWQG is not used by the province/territory;

The database was further analyzed to identify which parameters are consistently applied (at the same value) across all jurisdictions; which jurisdictions use different values for given parameters; which parameters are applied widely but not by all jurisdictions; which are the ‘outlier’ parameters, *i.e.*, parameters that are only applied by one or two jurisdictions.

Three researchers re-checked all the data in the database for accuracy. The data and analysis was then sent to relevant agencies in each of the ten provinces and three territorial jurisdictions to verify the data and analysis were correct to ensure that the inventory was both comprehensive and accurate. Agency staff that reviewed the data included Provincial Health Officers, Drinking Water Officers, water quality specialists, scientists and technicians. Over 90% of the provincial agencies contacted provided feedback. All but one jurisdiction responded with comments, which were subsequently incorporated into the analysis. 

## 3. Results

Guidelines, standards, codes of practice, and policies only become enforceable when they are implemented in legislation. Since the Canadian Drinking Water Quality Guidelines are voluntary non-enforceable guidelines, Canadian provinces and territories are able to choose: (i) whether or not to adopt the CDWQG; (ii) to what extent they apply the guidelines, and (iii) whether to make them legally enforceable [3].

### 3.1. Legal Approaches to Drinking Water

Eight of the 13 jurisdictions have legally binding drinking water regulations (see Table 1). However, only one of these eight jurisdictions, the Northwest Territories, has adopted all 94 CDWQG into provincial regulation. Nova Scotia and Manitoba have adopted the majority of the CDWQG into provincial regulation. The remaining five jurisdictions (Alberta, Saskatchewan, Ontario, Québec and Yukon) have partially adopted the CDWQG into legislation (see Table 2). 

Whilst BC follows the CDWQG in their entirety (see Table 2), only the microbial indicators are legally binding. According to the British Columbia Water and Wastewater Association (BCWWA), BC is currently the only provincial or state level of government in North America that does not explicitly identify drinking water standards beyond bacterial indicators. Non-legally binding approaches are also taken in New Brunswick, Prince Edward Island, Newfoundland and Labrador and Nunavut. 

**Table 1:** Legal enforcement of drinking water standards.

Jurisdiction	Legally Binding	Comments
British Columbia (BC)	N	Depending on the variable in question and the nature of the water supplier there may be an absolute requirement to meet a guideline, or there may be allowances for slight elevation above the guideline, or periodic exceedance.
Alberta (AB)	Y	Public Health Act—Nuisance and General Sanitation Regulation (Section 11) and the Environmental Protection and Enhancement Act (EPEA).
Saskatchewan (SK)	Y	The Saskatchewan Standards are enforceable under The Environment Management and Protection Act, 2002 and The Water Regulations, 2002.
Manitoba (MB)	Y	Chemical standards are enforced under the Drinking Water Quality Standards Regulation. Manitoba adopts all other parameters either from CDWQG or CCME as general guidelines by policy.
Ontario (ON)	Y	Safe Drinking Water Act, Ontario Regulation 169/03—Ontario Drinking Water Quality Standards.
Québec (QC)	Y	Environment Quality Act, under “Regulation respecting the quality of drinking water”.
New Brunswick (NB)	N	Although the NB guidelines are not in regulation (so are technically guidelines rather than standards), they are enforceable via two mechanisms; (1) Clean Environment Act and (2) Public Health Act (by Department of Health via the powers of the Medical Officers of Health to issue orders).
Prince Edward Island (PE)	N	Exceeding a guideline would not be the issue in itself; however, failing to act in a responsible manner to prevent exceeding a guideline would be an enforcement issue.
Nova Scotia (NS)	Y	“Health”-related parameters (microbiological, physical and chemical) are legally enforceable under the regulation. “Non-health” related parameters (aesthetic) are guidelines and not legally enforceable.
Newfoundland & Labrador (NL)	N	Depending on the situation, different pieces of legislation can be applied: (1) Guidelines enforced by placing the community on a non-consumption advisory if there is a “contaminant” or exceedance of a health based guideline, (2) A non-consumption advisory is issued under the Health and Community Services Act, which basically gives the officials the “power” to issue the advisory, (3) Subsection 39 (3) of the Water Resources Act gives the authority to take action to correct or prevent adverse affects to a public water supply.
Yukon (YK)	Y	Drinking water regulation: Public Health and Safety Act—Drinking Water Regulation .
Northwest Territories (NT)	Y	Adopted by the Water Supply System Regulations.
Nunavut (NU)	N	Aboriginal Affairs and Northern Development Canada (AANDC) is responsible for all water policy in Nunavut. It is unclear whether or not drinking water quality standards are legally binding in the territory.

It is however, important to note that this is a constantly shifting landscape. There have been a number of revisions included in the 2012 CDWQG since the previous version of the guidelines was published in 2010. Québec overhauled their drinking water quality legislation in June 2013 and the Saskatchewan government is currently reviewing and updating their regulations and plan to have new legislation shortly (our validation process confirmed the new regulations will be tied closely to the Health Canada CDWQG). BC is also in the process of updating its water regulations with new laws planned for 2014. 

### 3.2. Jurisdictional Application of the Canadian Drinking Water Quality Guidelines

In total, 127 unique drinking water parameters are used across the 13 Canadian provinces and territories, 94 of which comprise the Canadian Drinking Water Quality Guidelines. Table 2 summarizes the application of Canadian Drinking Water Quality Guidelines, by each province and territory. The table illustrates whether jurisdictions follow the guidelines (using the associated recommended values) or whether they apply more stringent, or less stringent values. The table also illustrates the use of parameters not listed in the CDWQG. 

**Table 2:** Use of CDWQG parameters in Canadian provinces and territories.

Jurisdiction	No. of Parameters Same as CDWQG (Out of 94)	No. of Parameters Less Stringent Than CDWQG	No. of Parameters More Stringent Than CDWQG	No. of CDWQG not Applied by Jurisdiction	No. of Parameters Applied by Jurisdiction, but not Listed in CDWQG	Total # Parameters Used by This Jurisdiction
British Columbia (BC)	94	0	0	0	0	94
Alberta (AB)	72	0	0	22	0	72
Saskatchewan (SK)	56	6	0	32	3	65
Manitoba (MB)	90	1	0	1	3	94
Ontario (ON)	79	3	3	9	21	106
Québec (QC)	36	2	35	21	10	83
New Brunswick (NB)	93	1	0	0	0	94
Prince Edward Island (PE)	94	0	0	0	0	94
Nova Scotia (NS)	93	1	0	0	2	96
Newfoundland & Labrador (NL)	92	0	1	1	1	94
Yukon (YK)	27	0	0	68	1	28
Northwest Territories (NT)	94	0	0	0	0	94
Nunavut (NU)	94	0	0	0	0	94

Four jurisdictions follow the CDWQG in its entirety: British Columbia, Prince Edward Island, Northwest Territories and Nunavut. As mentioned previously, these are only fully legally binding in the Northwest Territories. The Yukon and Québec utilize the fewest number of CDWQG’s (27 and 36 respectively) with the Yukon omitting 68 CDWQG parameters and Saskatchewan 32. Québec substantially revised their drinking water standards in June 2013, and this province now has the most stringent standards of all the jurisdictions (35 parameters which are more stringent than the CDWQG). Québec also applies standards for 10 parameters not listed under CDWQG (such as bromodichloromethane; however, six of these 10 parameters were previously listed under CDWQG but have since been archived). Ontario has three standards more stringent than the national guidelines, and 21 additional standards not currently listed in the CDWQG. Ontario has the most parameters of all the jurisdictions (106 in total), whereas the Yukon has the least (28 in total). 

One explanation for discrepancies between jurisdictions having guidelines not listed under the CDWQG (or more/less stringent values than the CDWQG) is that some have been archived or revised in subsequent updates by the Federal-Provincial-Technical Committee on Drinking Water. New Brunswick’s guideline for carbon tetrachloride offers a good example of this. The CDWQG has been updated to a new, more stringent value (0.002 mg/L), but New Brunswick is still quoting the old guideline (0.005 mg/L). They were recently made aware of this discrepancy and intend to revise it to align with the GCDWQ, but as of writing this has yet to be formalized. 

There are 18 “outlier” parameters *i.e.*, parameters that are only used in one or two provinces. Interestingly, none of the 18 outliers are listed under the CDWQGs. At least 10 of these parameters previously had a CDWQ guideline value; however, they have since been archived by the FPTCDW, which has determined that they no longer pose a threat to drinking water. These 18 outliers appear in only three jurisdictions. Ontario has the most outlier parameters (14 in total) followed by Saskatchewan and Ontario, which have two outliers each. Again, there may be multiple reasons for this, but it might simply be a reflection of the lag time for provinces to revise their standards and expectations—an issue explored further below with respect to some of the challenges with such a decentralized approach. 

### 3.3. The Canadian Baseline

There are only 16 Canadian Drinking Water Guidelines that all provincial and territorial jurisdictions apply uniformly (*i.e.*, using the same value). These 16 parameters are listed in Table 3 below. Effectively, these are the current “common baseline” for drinking water quality, which all provinces and territories share.

### 3.4. Microbial Water Quality

Most provinces and territories have, at a minimum, legally binding standards for microbiological parameters (usually two bacteriological indicators total coliforms and *E. coli*) [23,24]. New Brunswick and Prince Edward Island are the latest jurisdictions to introduce legally binding standards for drinking water quality including microbiological parameters [19,20,21]. Whilst PEI does not have explicit independent microbiological standards, testing for microbiological analysis is required [19]. However, a water utility’s operating permit may require them to test for additional microbial parameters that are not required in provincial legislation [26].

**Table 3:** Canadian Drinking Water Quality Guidelines—the common baseline.

Type	Parameter	CDWQG Value
Chemical and Physical	Barium	1 mg/L (MAC)
	Benzene	0.005 mg/L (MAC)
	Benzo[a]pyrene	0.00001 mg/L (MAC)
	Boron	5 mg/L (MAC)
	Cadmium (total)	0.005 mg/L (MAC)
	Chromium (total)	0.05 mg/L (MAC)
	Copper	≤1 mg/L (AO or OG)
	1,4-Dichlorobenzene	0.005 mg/L (MAC)
	1,2-Dichloroethane	0.005 mg/L (MAC)
	Dichloromethane (methylene chloride)	0.05 mg/L (MAC)
	Fluoride (total)	1.5 mg/L
	Lead	0.01 mg/L (MAC)
	Mercury Uranium	0.001 mg/L (MAC) 0.02 mg/L (MAC)
Microbiological	*E. coli*	0 E. coli per 100 mL
	Total coliforms	0 total coliforms/100 mL

The 2012 CDWQG now include guidelines for Giardia, Cryptosporidium, and enteric viruses, but (as shown in Table 4) a handful of provinces and territories have yet to formally embrace these newer standards. Notably, the United States has more extensive microbiological guidelines than Canada, which include Maximum Contaminant Level Goals (MCLGs) of zero for emerging contaminants *Cryptosporidium*, *Giardia lamblia*, *Leguinella* and enteric viruses. Indeed, *E.coli* remains the universal standard for microbiological parameters, but there is no equivalent for chemical parameters. “*Even though the overall guidelines package is reasonably careful to stress that microbial pathogens pose the greatest risk to human health from drinking water, there remains an inordinate focus on chemical hazards*” [2].

**Table 4:** Microbial drinking water quality guidelines.

Microbial Parameter	CDWQ Guideline	Jurisdictional Application
Total coliforms	0/100 mL	All provinces & territories
*E. coli*	0/100 mL	All provinces & territories
*Giardia*	Treatment goal: Minimum 3 log reduction and/or inactivation	Seven provinces: AB, MB, NL, NS, PEI, QC one territory: NWT
*Cryptosporidium*	Treatment goal: Minimum 3 log reduction and/or inactivation	Seven provinces: BC, AB, MB, NL, NS, PEI, QC; 1 territory: NWT
Enteric viruses	Treatment goal: Minimum 4 log reduction and/or inactivation of enteric viruses	Seven provinces: BC, AB, MB, NL, NS, PEI, QC; 1 territory: NWT
Enterococci	None required	Not applied

To summarize: we find substantial variation in the number of drinking water parameters being applied, with only 16 of the 94 CDWQG applied uniformly across all Canadian provinces and territories. There are 18 parameters that only a few jurisdictions use (none of these parameters are listed under the CDWQG). There is substantial variation in the strength of governance approaches, notably the distinction between mandatory and voluntary guidelines, with only eight of the 13 provinces and territories having legally binding drinking water standards. 

## 4. Discussion

Our research shows that the CDWQG are used in a variety of different ways across Canadian jurisdictions: either in their entirety or partially, as guidelines or legally binding standards. In short, the voluntary system of drinking water guidelines at the federal level has produced considerable heterogeneity at the provincial level both in terms of the application of drinking water quality parameters as well as the extent of legal enforcement. This research is supported by findings in Cook *et al.*, which highlighted significant provincial variation in regulatory approaches, including uneven compliance and enforcement, of microbial drinking water quality drawing on comparisons between BC, Ontario and Québec [26]. Our findings are also generally consistent with the fragmented landscape of water governance across the country [10,12]. Moreover, since it is the water providers that bear the primary responsibility to carry out water quality sampling we predict this variation will further continue at the municipal/local scale. For example, research by Dunn *et al.* indicates that some municipalities undertake additional water testing using parameters not listed under the CDWQG (e.g., enterococci bacteria) [27], whilst other water providers struggle to maintain sampling requirements [3].

What can be learned from the Canadian example, particularly given global interest in decentralized water governance? In what ways might Canada suggest cautionary tales for drinking water quality governance, or conversely, might Canada provide a model to be followed for other contexts seeking to minimize regulatory approaches and enable more local-scale responses better suited to variable conditions on the ground? In either scenario, a key consideration here is to assess and draw insights from the Canadian context to inform contemporary international debates. Here we discuss a number of issues currently debated in the literature, which are of direct relevance to interpretation of our results, including mandatory standards versus voluntary guidelines; harmonized versus delegated governance and uniform versus variable guidelines. 

### 4.1. Mandatory Standards vs. Voluntary Guidelines and System Knowledge

The World Health Organization (WHO) recommends that all countries have national legally binding drinking water standards [8]. The USA and the European Union, both of which share some similarities with Canada as federated systems, have adopted uniform and harmonized federal regulations (or, in the case of the EU, super-federal regulations) to ensure consistent standards across their respective territories. However, in Canada, in line with the constitution, jurisdictions have taken individual approaches to drinking water quality in both the parameters used and regulatory frameworks [1,3] with only four provinces using the CDWQG in their entirety and only eights provinces having mandatory standards. Some commentators argue that the Canadian voluntary approach has resulted in a fragmented, ad hoc approach across provinces and territories with varying degrees of adoption [2,9,12,14,17,28] creating a system prone to gaps and overlaps, which, for example, Christensen describes as “lax” [20]. In the Walkerton Inquiry report, Justice O’Connor recommended that drinking water quality standards have the force of law [29]. Prominent Canadian non-governmental organizations echoing the Walkerton Inquiry Report have similarly called for legally binding regulations at the national level arguing that the current approach is of serious concern [17,18,19,20,21]. 

It is clear that there is no consensus with respect to how to govern and regulate drinking water quality across large and geographically complex territories. Apart from mixed suggestions related to the value of harmonized, or devolved water governance, there are other related debates, which have implications for these issues. Among them, there is emerging trend in the academic literature that suggests a focus on compliance testing (relative to specific guidelines) is sub-optimal: it is reactive (identifying problems after they have occurred); monitoring and testing can be expensive, and can often be an ineffective use of limited funds. Greater emphasis is being placed on proactive, preventative multi-barrier approaches and developing institutional and “system knowledge” (*i.e.*, in-depth understanding of the water supply system from the source through to treatment and distribution) and capacity (institutional, financial, technical, human resources, social). Hrudey, for example, emphasizes the importance of well-trained and competent operators with in-depth knowledge and understanding of the water supply system (source, treatment and distribution). He argues that these skills should be supported by regional capacity, an important factor given economies of scales and pooling of resources. As Hrudey suggests “…*throwing money at a problem without having the money guided by those who understand what is required will achieve little, or may even be counter-productive*” [2]. In this vein, some commentators argue in favour of “light handed” regulations that facilitate regional variation and allow greater emphasis to be focused on achieving and demonstrating competence relative to the specific risks facing a system [2,3,16,28]. Arguably, more decentralized approaches are also generally consistent with neoliberalization processes, where responsibilities are increasingly downloaded to municipalities, territories, or state governments, with the idea that those closer to the ground, and far from bureaucratic centers of power, can manage more effectively, and with greater consideration or local realities on the ground (see Heynen *et al.*, [30] for a discussion of decentralization and similar trends as part of neoliberalized environmental governance, as well as recent trends to move towards watershed scale governance, as discussed in Cohen and Davidson [22], and as detailed further below).

Whilst there are costs, capacity tradeoffs, as well as tensions between regulatory and consumer expectations [3], our analysis nonetheless suggests that there are considerable gaps and inefficiencies in evidence in the Canadian context at present (several discussed in the following sections) and further investigation is required. Among them how decentralized approaches based on local system knowledge can proceed with broader systems of support (whether in terms of expertise, guidance, resources, or to ensure that all citizens are protected equally—all of which evidence from our work and the broader literature suggests are difficulties associated with fragmented and decentralized approaches)? Whilst we agree with Hrudey regarding the importance of system knowledge and competent well-trained operators at the local scale, we argue that this is not an either/or proposition. Our analysis implies that an overarching structure of drinking water quality standards/guidelines may be important, as it creates a higher or lower threshold of acceptable drinking water quality for which local operators are held accountable. In sum, a balance needs to be struck between adequately protecting drinking water safety through regulation, whilst accommodating spatial variation and considering capacity, particularly for smaller systems. 

These trends are part and parcel of the larger trend of decentralized approaches, in favor of locally adapted conditions, knowledge, and governance frameworks. Thus, while the arguments and rationales are different (some interested more in overcoming bureaucratic inefficiencies, others more in participatory democracy), in some ways a multi-barrier approach reinforces other trends towards devolution of drinking water approaches. While we cannot speak to these concerns in an comprehensive sense, our analysis nonetheless suggests that the variation we observe across Canada indicates that although the framework might be amenable to localized ‘system knowledge’, time lags in adapting requirements or regulations suggests considerable inefficiencies with such a fragmented approach. There are also other consequences, including lack of integrated data, and potential inequities, which we detail further below. 

### 4.2. Harmonized vs. Delegated Governance

We found 127 unique drinking water parameters being used across the 13 Canadian provinces and territories, 94 of which comprise the Canadian Drinking Water Quality Guidelines. The very fact that some drinking water quality parameters are being modified, supplemented and omitted by jurisdictions, rather than being automatically updated when the CDWQG raises a number of questions. Why do differences exist between the provinces and territories? Why does one jurisdiction deem one level safer than another and so forth? It could also be suggested that some resources might be used inefficiently—either maintaining standards that are no longer recommended, or failing to take on new requirements as they emerge and are considered important. Whilst it is clear that resource considerations may make requirements for monitoring and enforcement onerous for some, what are the risks and costs of maintaining such a patchwork approach? On the other hand however, it is clear that just as the CDWQG are modified to try to take into account emergent concerns, or updated scientific evaluation of concerns, there may be concerns more or less relevant to different jurisdictions dependent on biophysical or other elements. Here, there is clear benefit of not requiring binding standards across a territory as vast and variegated as Canada. However, it is possible that those risks thought to be more relevant for all territories could be described as such, while considerations more variable could also be considered options as part of a national assessment of concerns and standards (*i.e.*, perhaps “core” parameters could be identified, and mandated, with a complementary optional set of recommended parameters, more dependent on context specific requirements).

Another risk or outcome of the decentralized approach in Canada has been the impact on data collection and quality. In Canada, there is currently no national drinking water surveillance system, nor is there a standardized approach to collect drinking water quality information [21]. Data collection and methods vary from province to province and this heterogeneity renders data collection and data sharing difficult, if not impossible. Whilst there are many “pockets” of data (collected by various federal, provincial, municipal agencies and local interest groups), there is neither an overarching central surveillance or information retrieval system, nor consistency (or guidelines) in collection methods. For example, there is no systematic collection of water-borne disease outbreaks [31]. Limited data has impeded our ability to fully understand the impacts of burden of disease (including economic impacts); compare water quality between jurisdictions; and monitor national trends; this may produce what the OECD describes as “knowledge gaps” [32]. Based on the analysis conducted for this paper, we have made a minor contribution towards this considerable gap by providing our inventory of drinking water quality parameters used in the 13 Canadian provinces and territories (the first of its kind), on our website [33]. 

### 4.3. Uniform vs. Variable Guidelines

We find that only 16 of the 94 CDWQG are consistently applied across all 13 jurisdictions. There are obvious practicalities that would make testing of all parameters a significant challenge if the guidelines were made mandatory, namely the financial and staff implications. Moreover, the real benefit of such an approach is unclear. Variation, however, also runs the risk of exacerbating inequities. The current situation in Canada is one that many observers have noted is highly inequitable, in terms of variable standards and approaches. Here we highlight two pressing Canadian concerns documented in the literature: (i) urban-rural disparities and (ii) poor water quality in First Nations communities. We bring these up as part of our discussion to further explore the consequences of variability, not just those that exist between provinces and territories, but also how this filters down and can be further exacerbated at municipal and local scales. Together, these concerns speak to the problems inherent when a uniform approach is not applied—leading to a patchwork of approaches and with the threat of exacerbating inequalities. 

#### 4.3.1. Small Drinking Water Systems

Whilst responsibility for drinking water quality in Canada may be shared between the provinces, health authorities and municipalities, it is largely the municipalities that bear the burden of responsibility to provide safe drinking water [3,34]. Given this, it is clear that the variable approach we have documented at the provincial scale is exacerbated when more local systems are taken into account. Indeed, the literature suggests that there is growing disparity between larger and smaller (mostly rural) systems in Canada [21,35,36,37] with larger municipalities typically test for more drinking water parameters than smaller systems. “*Larger systems are held to more rigorous standards as those systems are generally able to purchase better technology and hire specialized personnel*” [38]. In contrast, smaller systems typically have fewer resources and less capacity (including financial, technical, staff and institutional), fewer monitoring requirements; may lack the financial capacity to build extensive water treatment facilities [39]; have fewer highly trained personnel [3,35,36,37]; and limited capacity to meet sampling requirements [3]. Shortfalls in the technical, managerial and financial capacities have been contributing factors in major waterborne disease outbreaks including the one that occurred in Walkerton in 2000 [37,40]. Each province has responded differently to the challenge of managing water quality in smaller systems. For microbial water quality for example, Ontario has created a separate regulatory framework for small drinking water systems; British Columbia has exemptions for smaller systems, as has Québec for systems serving less than 21 persons [26]. Some commentators have argued that as such, smaller communities are at higher risk and are more likely to experience an outbreak of infectious disease than people receiving their water from a large public utility [3,5,35]. For example, BC currently has over 530 Boil Water Advisories in effect (the highest per capita in Canada), the majority of which are on small systems [41]. 

Of course, stricter regulations also pose a problem: without adequate resources, this risks becoming an “unfunded mandate”, creating significant problems for small communities. Rural communities are substantial in number, but fragmented and dispersed across the country making these systems hard to monitor, particularly communities that are hard to reach [35,36]. Some commentators suggest that these disparities have effectively created “*two-tier system of supply roughly split along the urban-rural divide*” [21,35]. Cook *et al.*, suggest unique regulatory approaches for systems of different sizes may be necessary to resolve the challenges small water systems have in meeting water quality requirements designed for larger systems [26]. However, our analysis suggests that the absence of a baseline, uniform and regulated approach means that different populations are effectively exposed to different levels of risk—a classic environmental justice concern, as often it is lower income or vulnerable populations that may not enjoy adequate levels of protection.

#### 4.3.2. Poor Drinking Water Quality on First Nations Reserves

Linked to the urban-rural issue is drinking water quality on First Nations reserves. The reasons for this differential are not exactly the same as those that affect rural systems, but again, are strongly linked to differential governance pathways and expectations. Patrick argues, “*Access to safe drinking water in Canada is a function of both where you live and who you are*” [4]. A 2008 study in the Canadian Medical Association Journal showed that there were 93 Boil Water Advisories (BWAs) in First Nations communities, many of which are long-term, with some in effect for more than 10 years. “*The number of water-borne infections in First Nations communities is an alarming 26 times higher than the Canadian national average*” [1,4]. Currently, First Nations drinking water quality does not fall under provincial jurisdiction. Rather, it is the requirement of federal systems to work with First Nations to ensure drinking water quality on reserves—a system which many have argued have left many of these reserve communities as falling between the cracks, given lack of provincial involvement and investment. O’Connor’s recommendations for greater regulation and uniformity included a call for drinking water standards to be applied to reserves [9,29]. A process is underway to transition drinking water quality on First Nations from federal to provincial/territorial jurisdiction. Given the considerable variation that exists between provinces and territories, our analysis suggests that this shift will not overcome the variable conditions related to drinking water quality and protection for First Nations. Thus, while further provincial involvement may be beneficial in many cases, there is also the risk that variation across the provinces means that the situations of reserves will remain highly unequal. In effect, while federal mandates for First Nations drinking water management and protection have clearly not been effective, this would replace it with a system where reserves may still face less protection as compared with larger municipal systems, and also with key differences between reserves located in British Columbia compared to those in Alberta, for instance. There is reason for particular concern in provinces where there are generally fewer drinking water quality standards (see Table 2), as we are likely to see significant variation at sub-provincial scales. 

In sum, differential access to resources for monitoring, enforcement, and treatment has the potential to further exacerbate an already fragile (and largely failing) approach. While the current situation is clearly flawed—it also seems likely that without more significant governance reform, these issues will likely remain significant challenges, and significant concern, into the future. This highly unequal situation also suggests that any attempt to reform governance processes to more adequately deal with drinking water challenges needs to foreground the particular needs and challenges of First Nations. These issues are important to highlight, we suggest, given that these types of inequities are key to any discussion of whether decentralization or harmonization is likely to be effective, or to consider in what ways we might more adequately deal with drinking water challenges. First Nations water governance raises issues about co-governance, rights and title, and other complexities. However, at the core, we also see the broader issues of variable access to resources, and key inequalities, which are directly connected to broader debates related to debates related to decentralized approaches to drinking water regulation, monitoring, and management. 

## 5. Conclusions

This study analyzed the uptake of the voluntary Canadian Drinking Water Quality Guidelines across Canada’s 13 jurisdictions (provinces and territories). Jalba *et al.* identified unclear and/or flawed drinking water regulatory regimes as a major governmental failure and a contributing factor to drinking water incidents [40]. Canada provides an interesting case study in this regard, given that its approach to drinking water regulation is arguably the most decentralized of any OECD country. In this paper, we have argued that the Canadian approach to drinking water standards and guidelines has resulted in data gaps, urban-rural, and other disparities. While certain elements of this variability may be beneficial (e.g., enabling for greater system knowledge to deal more adequately with risks in a source-to-tap framework), it is also clear that variability in approaches and requirements poses considerable challenges that need to be taken seriously in designing more effective multi-level governance approaches (e.g., nested approaches that enable local flexibility, but within constraints and within a well specific framework providing expertise and guidance). In all of these ways, we can learn from cases such as Canada, regarding the potential risks and opportunities of highly decentralized governance. Yet, further research is required regarding risks and opportunities of such approaches—including the potential public health implications, particularly with respect to small, rural, and other at-risk or underserved communities. In particular, this research raises a number of important questions for future research, relevant to those engaged in delegated water governance processes. Specifically, the analysis raises a key issue with respect to decentralization: should it include delegation of drinking water standards; and, if so, what are the implications? Variation raises some interesting conceptual issues for broader debates related to “minimum regulatory requirements”, including ideas about subsidiarity, equity, “system values” and the implications for public health governance [3,35,36]. Our analysis suggests that even with some possible benefits of decentralized approaches, such as flexibility in drinking water provision and management, oversight in order to maintain equity and public health goals remains a key concern.

Since water providers have the primary responsibility to carry out water quality sampling we recommend that further research be undertaken to determine the uptake of federal and provincial drinking water guidelines at the municipal/local scale. We also recommend that more research regarding the implications of our findings for the safety of drinking water in rural and First Nation communities is needed, including the potential public health implications, particularly for small and remote communities. However, we note that such research will be challenged by limited access to, and comparability of data within and between jurisdictions.

Further research on appropriate policy responses is also required. Increasingly, experts are calling for the federal government to take more of a leadership role in the overarching approach to water management [3,28]. The need for a degree of harmonization even in highly decentralized settings merits further study. A flexible approach may be successful, but likely only if there is a clear, consistent framework in which roles and responsibilities are clearly defined and facilitate positive outcomes, while mitigating or preventing potential negative outcomes from place-based variation in drinking water quality regulation, as signaled in the recommendations of the Walkerton Inquiry.

Highlighting the equity concerns also noted above, particularly specific to the complex governance issues for First Nations water quality, we also consider that much more work is needed with the goal of improving the situation on reserves. The current situation is clearly lacking, and new steps need to be taken to address these concerns. While the issue of First Nations drinking water quality is not the same as other equity concerns, we see this situation as highlighting the reason to foreground equity considerations in any debate related to harmonization or subsidiarity.
